# Distribution and Clonality of drug-resistant tuberculosis in South Africa

**DOI:** 10.1186/s12866-021-02232-z

**Published:** 2021-05-28

**Authors:** Halima Said, John Ratabane, Linda Erasmus, Yasmin Gardee, Shaheed Omar, Andries Dreyer, Farzana Ismail, Zaheda Bhyat, Tiisetso Lebaka, Minty van der Meulen, Thabisile Gwala, Adeboye Adelekan, Karidia Diallo, Nazir Ismail

**Affiliations:** 1 Centre for Tuberculosis, National Institute of Communicable Diseases, 1 Moderfontein Road, Sandringham, Johannesburg, 2131 South Africa; 2 Department of Medical Microbiology, Faculty of Health Science, University of Free State, Bloemfontein, South Africa; 3 Division of Public Health Surveillance and Response, National Institute of Communicable Diseases, Johannesburg, South Africa; 4 PathCare, Vermaak, Pretoria, South Africa; 5 Centers for Disease Control and Prevention, Pretoria, South Africa; 6 Department of Medical Microbiology, Faculty of Health Science, University of Pretoria, Pretoria, South Africa

**Keywords:** RR-TB, Genotyping, Spoligotyping, 24-loci MIRU-VNTR typing

## Abstract

**Background:**

Studies have shown that drug-resistant tuberculosis (DR-TB) in South Africa (SA) is clonal and is caused mostly by transmission. Identifying transmission chains is important in controlling DR-TB. This study reports on the sentinel molecular surveillance data of Rifampicin-Resistant (RR) TB in SA, aiming to describe the RR-TB strain population and the estimated transmission of RR-TB cases.

**Method:**

RR-TB isolates collected between 2014 and 2018 from eight provinces were genotyped using combination of spoligotyping and 24-loci mycobacterial interspersed repetitive-units-variable-number tandem repeats (MIRU-VNTR) typing.

**Results:**

Of the 3007 isolates genotyped, 301 clusters were identified. Cluster size ranged between 2 and 270 cases. Most of the clusters (247/301; 82.0%) were small in size (< 5 cases), 12.0% (37/301) were medium sized (5–10 cases), 3.3% (10/301) were large (11–25 cases) and 2.3% (7/301) were very large with 26–270 cases. The Beijing genotype was responsible for majority of RR-TB cases in Western and Eastern Cape, while the East-African-Indian-Somalian (EAI1_SOM) genotype accounted for a third of RR-TB cases in Mpumalanga. The overall proportion of RR-TB cases estimated to be due to transmission was 42%, with the highest transmission-rate in Western Cape (64%) and the lowest in Northern Cape (9%).

**Conclusion:**

Large clusters contribute to the burden of RR-TB in specific geographic areas such as Western Cape, Eastern Cape and Mpumalanga, highlighting the need for community-wide interventions. Most of the clusters identified in the study were small, suggesting close contact transmission events, emphasizing the importance of contact investigations and infection control as the primary interventions in SA.

**Supplementary Information:**

The online version contains supplementary material available at 10.1186/s12866-021-02232-z.

## Background

South Africa (SA) carries a disproportionate burden of drug-resistant tuberculosis (DR-TB) in Africa. The burden of DR-TB is largely driven by transmission [1–5]. Several studies in SA have reported a high-level of clonal DR-TB transmission [6–8]. Hence, understanding transmission dynamics of DR-TB remains critical in controlling this epidemic in SA.

Genotyping of *M. tuberculosis* strains has proven to be a powerful surveillance tool for understanding the transmission dynamics of TB. Several genotyping techniques have been developed to investigate population structure and transmission of *M. tuberculosis*. Insertion sequence IS*6110*-based restriction fragment length polymorphism (RFLP) analysis was considered the gold standard [9]. However, high-throughput polymerase chain reaction (PCR)-based methods have been developed, providing equivalent resolution. These include: spoligotyping [10] and mycobacterial interspersed repetitive-units-variable-number tandem repeats, (MIRU-VNTR) [11]. Of recent, molecular characterization using whole genome sequencing (WGS) is increasingly being performed with high discriminatory power but is costly and less standardized [12, 13].

Although several clonal outbreaks were reported in SA, knowledge regarding the DR-TB population and transmission at a national level is limited. Currently, genotyping results are not routinely used for TB control in SA. Genotyping is primarily used for research purposes in selected population risk groups and in limited geographic areas. Thus, there is a need to undertake broader molecular epidemiological surveillance of DR-TB in SA to describe the DR-TB population and identify transmission events.

In 2014, the Center for TB (CTB), at the National Institute for Communicable Diseases (NICD), in Johannesburg, established the first sentinel molecular surveillance of Rifampicin-Resistant-TB (RR-TB) in SA in order to determine the prevalent RR-TB strains in specific provinces and the extent of RR-TB transmission. RR-TB instead of all TB was chosen based feasibility and cognizant that detection of RR-TB had improved with the introduction of the Xpert MTB/RIF assay as the initial diagnostic test in SA. In the current study we report the RR-TB strain population in selected SA provinces/districts and the estimated proportion of RR-TB transmission.

## Result

Over the study period, 3007 culture confirmed RR-TB cases had genotyping results by both methods. Of these, 897 (29.8%) were collected from Western Cape (WC), 723 (24.0%) were from Eastern Cape (EC), 435 (14.5%) were from Mpumalanga (MP), 358 (11.9%) were from North West (NW), 230 (7.6%) were from KwaZulu-Natal (KZN), 142 (4.7%) were from Gauteng (GP), 135 (4.5%) were from Northern Cape (NC) and 79 (2.6%) were from Free State (FS). For eight (0.3%) isolates, no information on province was available. Culture negative samples and isolates without genotyping results by both methods were excluded.

### Strain lineages and diversity

Based on the spoligotype classification, 92.7% (2789/3007) could be assigned into previously described Shared International Type (SIT) types, 1.8% (55/3007) could be assigned to a lineage without SIT, while 5.4% (161/3007) isolates could not be assigned to any lineage (Table 1). The calculated genotype diversity varied by province, with highest in FS (18.9%) followed by NC (18.7%), GP (13.6%), KZN (10.3%), NW (8.3%), MP (5.9%), EC (3.4%), and WC (2.8%).

**Table 1:** Spoligotyping families of RR-TB cases prevalent in the different provinces of South Africa (2014–2018)

Family	SIT Identified	Provinces (No)									Total No (%) cases
		WC	EC	MP	NW	KZN	GP	NC	FS	Unknown province	
**Beijing**	1	599	517	125	88	57	44	37	26	2	1495 (49.7)
**LAM**	4,20,33,42,59,60,95,111,130,211,452,811,815,1607,1624,1873, No ST	53	55	47	54	42	29	31	17	1	329 (10.7)
**EAI**	6,48,236,702,806,947,1062,1251,1649	6	16	142	24	6	12	1	1		208 (6.9)
**T**	37,39,50,51,52,53,73,131,136,144,149,154,156,167,196,205,254,281,291,334,358,273,628,713,719,732,784,926,966,1067,1107,1129,1166,1332,1767	61	43	30	60	29	18	16	12	2	271 (9.0)
**X**	18,70,91,92,119,137,200,336,347,449,No ST	88	28	33	27	14	9	8	4	1	212 (7.1)
**S**	34,71,466,494,789,1211,1333, No ST	23	17	16	37	49	9	16	5		172 (5.7)
**H**	36,47,50,62,99,218,1822, No ST	10	7	15	26	8	6	9	5	1	87 (2.9)
**MANU**	54,100,226,124,714,800,	0	9	7	6	2	3	2	1		30 (1.0)
**CAS**	21,26,428,1092	8	4	0	6	5	2	3	2		30 (1.0)
**U**	2,384,584,231,907	7	0	2	1	1	0	0	1		12 (0.4)
**Unknown/Orphan**		42	27	18	29	17	10	12	5	1	161 (5.4)
**Total**		897	723	435	358	230	142	135	79	8	3007

**Fig. 1:**
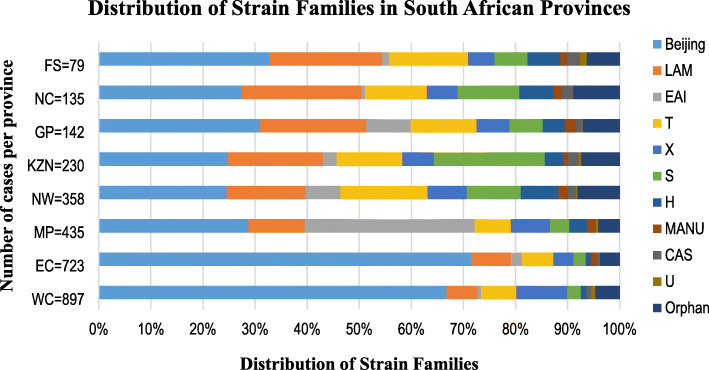
Distribution of RR-TB families in South Africa by province (2014–2018). FS, Free State; NC, Northern Cape; GP, Gauteng; KZN, KwaZulu-Natal; NW, North West; MP, Mpumalanga; EC, Eastern Cape; WC, Western Cape; Orphan, unknown genotype

The distribution of strain family stratified by province is shown in Fig. 1 and Table 1. The Beijing family strongly predominated in EC and WC accounting for 71.5% (517/723) and 66.8% (599/897) of the RR-TB isolates, respectively. The prevalence of Beijing was relatively lower in the remaining provinces, ranging between 22.9–32.9%.

The Latin American and Mediterranean (LAM) family was the second most prevalent genotype in five of the eight provinces, representing 22.9% of the strains in NC, while it has half of that in MP (10.7%). The LAM family was least prevalent in EC (7.6%) and WC (5.6%). The prevalence of S was highest in KZN (21.3%) and was mainly represented in this study by two SITs: SIT34 (67%) and SIT71 (15%). Whereas X (mainly X1) was highest in WC (9.8%) and was also detected in MP, KZN, GP, and NC, but occurred at lower frequency (5.6–7.6%) (Table 1).

The East-African-Indian (EAI) was particularly prevalent in MP, accounting for 32.6% (142/435) of all isolates. In GP and NW, the prevalence of EAI was notable, accounting for 8.4 and 6.7%, respectively. However, it was much lower (< 3%) in the remaining provinces. The EAI family in this study was mainly represented by sub-lineage East-African-Indian-Somalian (EAI1_SOM) (179/208; 86.1%). The T (mainly T1) was common in all the provinces, with prevalence between 11 and 15% in NW, GP, FS, KZN and NC while it was half of that in MP, EC and WC (5–7%). The Haarlem (H) family seemed to be more prevalent in NW and FS at ~ 6%, while it was much lower in KZN (3%), GP (2.8%) and WC (1.1%). The CAS, U, and MANU were the least prevalent genotypes.

### Cluster size and cluster frequency

**Fig. 2:**
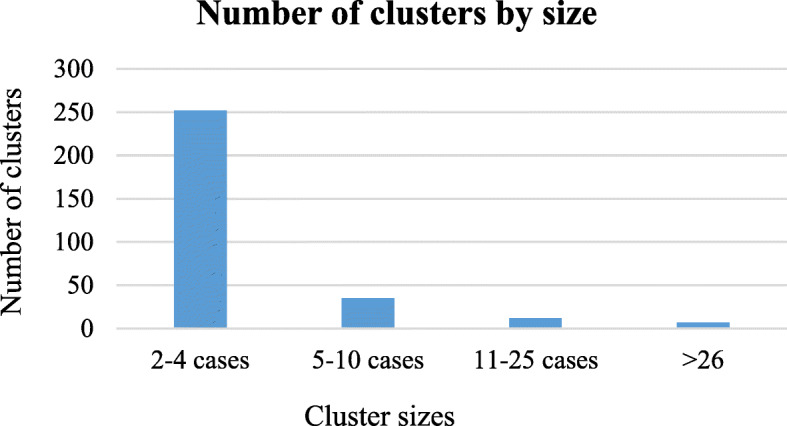
Number of clusters by size in South Africa (2014–2018)

Almost half of the isolates from surveillance sites, (52.2%, 1571/3007) belonged to molecular clusters and 1436 (47.8%) had a unique pattern. There was a total of 301 clusters, ranging between 2 and 270 cases. Most clusters (247/301; 82.0%) were small (< 5 cases), 12.3% (37/301) were medium sized (5–10 cases), 10/301 (3.3%) were large (11–25 cases) and 2.3% (7/301) were very large with 26–270 cases (Fig. 2). When analyzed by province, 89/301 (29.6%) clusters were from WC (2–262 cases/cluster), 64/301 (21.3%) clusters were from EC (2–100 cases/cluster), 48/301 (15.9%) from MP (2–43 cases/cluster), 42/301 (13.9%) from NW (2–8 cases/cluster), 28/301 (9.3%) from KZN (2–13 cases/cluster), 15/301(5.0%) from GP (2–9 cases/cluster), 10/301 (3.3%) from NC (2–3 cases/cluster), and 5/301 (1.7%) from FS (2–4 cases/cluster).

Using the n-1 method, the estimated overall transmission-rate for the study was 42%, with the highest in WC (64%) and the lowest in NC (9%) (Table 2). The clusters with 11–25 cases (9/10 clusters) and ≥ 26 cases (seven clusters) were found within three provinces (WC, EC and MP). WC and EC each had three clusters with ≥26 cases. The majority of the clusters from other provinces were small (< 5 cases), with few medium clusters from NW, KZN and GP (Table 2).

**Table 2:** Estimated transmission rate by provinces/districts (2014–2018)

Provinces	No. of isolates	No. of cases clustered* n	No. of clusters	Estimated proportion of cases likely due to recent transmission %	Number of clusters by cluster size			
					2–4 cases	5–10 cases	11–25 cases	≥26
WC	897	661	89	64	68	13	5	3
EC	723	435	64	51	47	11	3	3
MP	435	193	48	33	42	4	1	1
NW	358	116	42	21	38	4		
KZN	230	90	28	27	23	4	1	
GP	142	41	15	18	14	1		
NC	135	22	10	9	10			
FS	79	13	5	10	5			
Total	2999	1571	301	42	247	37	10	7

*n* number, *WC* Western Cape, *EC* Eastern Cape, *MP* Mpumalanga, *NW* North West, *KZN* KwaZulu*-*Natal, *GP* Gauteng, *NC* Northern Cape, *FS* Free State

The estimated transmission-rate for strain families identified in the study is shown in Table 3. Beijing family had the highest transmission rate (64.2%) followed by X (45.8%) and EAI (42.3%).

**Table 3:** Estimated transmission rate by strain family (2014–2018)

Family	No. of isolates	No. of cases clustered* n	No. of clusters	Estimated proportion of cases likely due to recent transmission %
Beijing	1495	1096	136	64.2
X	212	122	25	45.8
EAI	208	106	18	42.3
LAM	329	146	38	32.8
H	88	28	4	27.3
S	172	64	24	23.3
T	271	80	21	21.8

*n* number

The distribution of clusters with greater than four cases/cluster is shown in Table 4. Beijing family showed the highest clustering, especially in EC and WC. The two largest clusters (containing 113 and 270 isolates/cluster) identified in this study belonged to Beijing family. The cluster with 113 isolates was mostly detected in EC (100/113 cases), with only few cases in FS, MP, KZN and NW. Other Beijing clusters (containing between 5 and 65 isolates/cluster) were also identified in EC (Table 4). The Beijing cluster with 270 isolates was mostly (262 isolates/cluster) found in WC, with few isolates from EC, GP, MP and KZN (Table 4). The cluster was identified in all three districts of WC, with majority from City of Cape Town (199/262, 76%), followed by Cape Winelands (42/262, 16%) and West Coast (19/262, 7.3%). The Beijing clusters in the other provinces were small (2–4 cases) with few exceptions (Table 4).

**Table 4:** Distribution of clusters with greater that four cases in South Africa by provinces (2014–2018)

Family	SIT	24-loci MIRU-VNTR cluster	Cluster size	WC	Number of cases per province					NC	NW
					EC	FS	GP	KZN	MP		
Beijing	1	234,233,352,844,425,173,453,823	270	262	1		2	2	3		
		235,213,342,844,425,163,353,933	113		100	2		3	2		6
		244,232,352,644,425,173,354,624	65		60			2	2	1	
		235,213,342,644,425,163,353,933	55		45	4	2	1	1	1	1
		244,232,352,644,425,173,354,624	44	44							
		235,213,342,834,425,163,353,933	21		14	1			3	1	2
		244,233,352,242,425,173,353,824	17	17							
		244,232,352,644,425,174,354,624	16		16						
		234,233,352,444,425,173,453,823	15		1		2	4	8		
		244,232,352,644,425,174,356,624	14		13				1		
		244,232,352,644,425,193,356,622			7		1	3	1		2
		244,233,352,844,425,183,353,823	12	12							
		235,213,342,644,425,163,353,933		12							
		244,232,352,644,425,174,354,624	11	11							
		2,352,133,426(8)44425163353933	10		9	1					
		235,213,342,744,425,163,353,933			7	1	1		1		
		244,232,352,544,425,174,354,624	9		4			2		3	
		244,233,352,844,425,183,356,223			7	1				1	
		234,233,352,444,425,173,453,223	8		1		1	6			
		244,232,352,444,425,173,354,624	7		7						
		244,232,352,644,425,173,354,224			3			1		2	1
		244,233,352,644,425,183,353,823			4		2				1
		245,233,252,844,425,173,356,823			1		3		2		1
		235,213,342,834,425,123,353,933		7							
		244,233,352,644,425,182,353,823		7							
		244,232,352,644,425,172,354,624	6	6							
		235,213,342,644,425,163,353,133			5				1		
		244,213,352,444,425,171,353,823					2		3		1
		244,233,352,544,425,174,353,823					1	2	1	1	1
		244,233,352,844,425,174,356,823			1				3		2
		244,232,352,644,425,193,354,622	5		4		1				
		244,233,352,544,425,173,346,823			5						
		244,233,352,544,425,174,356,823						1	1	1	2
		244,233,352,844,425,183,353,223			5						
		235,113,342,644,425,163,353,832		5							
		2,352,133,426,444,251,633,531,133		5							
		244,033,352,544,425,173,353,823		5							
		244,223,352,644,425,183,353,823		5							
		244,232,352,544,425,173,354,624		5							
		244,233,352,544,425,174,353,823		5							
		244,233,352,544,425,183,353,823		5							
EAI1_SOM	48	2,225,342,112,393,246,223,347,211	48		1		4		43		
		222,534,242,393,246,223,347,211	17		2				12		3
		2,225,342,112,393,246,223,346,211	6		1				5		
		2,225,342,112,393,246,223,347,111	5		1				3		1
LAM4	60	134,244,332,224,126,153,332,832	31		2		9	13	2	3	1
		134,244,332,224,126,153,332,232	13		3			5	2	1	2
LAM3	33	243,234,342,324,226,153,131,722	15	10	1						4
		244,234,342,424,226,153,136,722	7		1	3		1		1	1
		244,234,342,424,226,153,131,722	5		2					1	2
X1	119	243,224,332,334,425,153,333,732	28	28							
X3	92	243,244,332,234,425,153,334,932	15		2			4	9		
		243,244,332,334,425,153,334,832	13		4	1	2		2	1	3
		253,254,332,333,425,153,323,832	8	8							
		243,244,332,334,425,153,224,832	6								6
		243,247,432,334,424,153,335,832	5						5		
H3	50	223,245,432,234,425,183,333,732	10		2		2	2	4		
H	N/A	123,233,332,834,425,153,333,632	11					1	1	1	8
T1	53	224,243,122,334,215,153,335,222	8		4				2		2
	719	244,234,342,424,226,153,136,222	7		2					1	4
	53	224,243,122,234,225,153,331,422	6		4		2				
S		324,343,212,324,225,143,336,222	8		1		1	4	2		
		324,343,212,824,225,143,336,222	6		1			3	1		1
Unknown genotype	N/A	243,244,332,334,425,153,334,832	5	5							

*SIT* Shared International *Type*, *WC* Western Cape, *EC* Eastern Cape, *FS* Free State*, GP,* Gauteng, *KZN* KwaZulu*-*Natal, *MP* Mpumalanga*, NC* Northern Cape, *NW* North West

In contrast, the EAI_SOM sub-lineage showed higher clustering in MP. The majority of the EAI_SOM clusters (11/15 clusters) were identified in MP, with the largest cluster containing 43 cases (Table 4).

Some clusters were specific to a certain province. Fifteen clusters of Beijing (contained 5–44 cases) were found only in WC, while three clusters (contained 5–16 cases) were found in EC. The X clusters (X1 [28 cases/cluster] and X3 [8 cases/cluster]) and a cluster with unknown genotype (5 cases/cluster) were exclusively found in WC. While two other X3 clusters containing five and six cases were identified in MP and WC, respectively (Table 4).

The LAM clusters were relatively small. Only five clusters had more than four cases including: LAM3 (contained 15, 7 and 5 cases/cluster) and LAM4 (contained 31 and 13 cases/cluster) sub-lineages. The two clusters of LAM4 differed with only one loci. The majority of cases for the cluster (*n* = 31) were from KZN (*n* = 13) and GP (*n* = 9), while the LAM3 cluster with 15 cases was mostly detected from WC (*n* = 10) and NW (*n* = 4).

## Discussion

The present study reports the first analysis of sentinel surveillance data on the distribution of RR-TB lineages in SA and transmission. The population structure of RR-TB isolates was dominated by Lineage 2 (Beijing) and Lineage 4 (Euro-American: LAM, T, S and X) strains. These patterns in genotype distribution likely reflect historical movement of strains. SA was located in a geographically central position in the historical trade route between East and West for hundreds of years, explaining the dominance of the Beijing (Eastern origin) and Euro-American strains (European origin) strains in SA [14].

The surveillance data showed geographic variation in RR-TB genotype distribution, which was consistent with previously published studies [15, 16]. WC and EC regions showed highly homogeneous strain population, with Beijing genotype representing majority (67 and 71%, respectively) of the RR-TB isolates. Previous studies in EC and WC reported that the Beijing strains account 54–69% of multidrug-resistant (MDR) and extensively drug-resistant (XDR) TB isolates [15, 17]. Interestingly, in MP the EAI (mainly EAI1_SOM) was the predominant genotype, representing a third of RR-TB cases. The EAI, however, was underrepresented in most of the other provinces (< 3%), with the exception of GP (8.2%) and NW (6.2%). This is in agreement with a previously published study that showed EAI1_SOM as predominant genotype in MP and GP [18]. Chihota et al. [15] also reported a higher prevalence of EAI1_SOM in GP compared to WC, EC, and KZN Provinces.

In contrast, RR-TB was caused by diverse genotypes in the remaining provinces, with predominance of five families (Beijing, LAM, T, S and X). The Beijing family represented the majority of RR-TB cases (22–32%). The LAM family was also common in all the provinces. The LAM family is prevalent in Latin-America and the Mediterranean regions as well as African countries such as Zambia and Zimbabwe [19, 20]. In SA, it is particularly prevalent in WC and KZN provinces [2, 15, 21]. The LAM3 (F11), is one of the endemic strains among drug-susceptible TB cases in WC [22], while the LAM4 (F15) has been reported as predominant strain among M/XDR-TB cases in KZN [2, 23–25]. In our study, the LAM was least prevalent among RR-TB cases in WC, suggesting strain variation among drug-susceptible and DR-TB population. In addition, differences in the distribution of the LAM sub-lineages was observed in this study. The LAM3 was more common in FS (12.6%), NW (8.1%), and NC (6.7%), while LAM4 sub-lineage has a higher representation in KZN (14.3%) and GP (10.6%). The frequent movement of KZN residents to GP, might explain the prevalence of LAM4 family in GP compared to other provinces.

The prevalence of T family (mainly T1) was notable in all provinces. The T family is one of the predominant genotypes reported in Africa [19, 26]. However, a lower proportion of this lineage was noted in MP, EC and WC (5–7%). The distribution of S was between 2 and 21% across the provinces, with highest prevalence in KZN and lowest in WC. The S family was previously reported to be prevalent in Algeria and to a lesser extent in SA, Madagascar and Egypt [19]. A study in KZN found S as predominant family among MDR-TB isolates collected between 2005 and 2006 [21]. The proportion of X (X1) lineage was higher (9.8%) in WC. Similarly, a higher prevalence of this family was reported previously in WC and NC provinces [27].

Cluster analysis showed almost half (42%) of the RR-TB cases in the selected districts in SA were due to recent transmission. The highest transmission-rate was found in WC (64%) and the lowest in NC (9%). Most of the clusters had 2–4 cases (82.0%) and likely represent small close contact transmission. The few large clusters (≥26 cases, 2.3%) identified in WC, EC and MP probably represent community transmission. However, it should be considered that clustering is not always indicative of recent transmission, as it can also reflect the persistence of endemic strains.

The majority of large and very large clusters were found in at least two province/district. However, some of the Beijing and X clusters were specific to a certain province/district (Table 4). This may be explained by the lack of strain exchange between geographically separated populations resulting in localized transmission.

Beijing family showed the highest (64.2%) clustering rate in this study. It is reported that the Beijing lineage appears to be more transmissible than other lineages [28]. The two largest clusters belonged to Beijing family and were found in five provinces, with most cases from WC and EC. The cluster with 113 cases corresponding to atypical Beijing strain [29] was mostly detected (100/113 cases) in EC. Previous study reported that the atypical Beijing strains are over-represented among RR-TB strains in EC [17]. This genotype was detected previously (2008) in 50% of RR-TB isolates in EC and might have gained a selective advantage over other strains to spread in the community [1, 15]. The atypical Beijing, however seem to be less prevalent worldwide with exception of Japan, Vietnam and Taiwan [30–34]. Conversely, the Beijing cluster with 270 isolates was mostly (262) found in WC, with majority (75.5%) of the isolates being from City of Cape Town. The presence of large Beijing clusters may indicate successful circulation of the lineage within the population. Nearly 80% of the reported MDR-TB cases in WC are due to primary transmission [35–37]. The distinct Beijing population in EC and WC may be a result of geographically localized outbreaks with limited strain exchange between the two regions. Previously conducted study showed that, 75% of MDR isolates of the Beijing genotype in WC belonged to typical Beijing strains, while 92% of the Beijing genotype in EC belonged to atypical Beijing strains [15].

The X lineage showed the second highest clustering rate (45.8%) in this study. It is one of the predominant strain families in the WC. The X1 cluster containing 28 isolates and X3 cluster with eight cases were identified in WC. The X strains have been reported to have caused large DR-TB outbreaks in WC historically [8, 38].

The estimated transmission-rate for MP in this study was relatively high (33%). The transmission of RR-TB in MP seem to be driven by EAI lineage, showing the clustering rate of 42.3%. The two large EAI1_SOM clusters were mostly detected in MP (12/17 and 43/48 case) (Table 4). The EAI strains are reported to be prevalent in neighbouring country Mozambique, and east African countries such as Sudan, Djibouti, Malawi, and Madagascar [19]. The EAI may be introduced to MP from Mozambique through cross-border movement, as there is high Mozambican migration to SA in search of better economic opportunities [39]. Unlike Beijing and LAM, the EAI seem to be geographically restricted to MP, with limited transmission to GP and NW. The reasons for this geographically restricted transmission may have to do with adaptation of the strain to specific population in that geographical setting and/or it could be due to the low transmissibility of EAI lineage as compared to the other strains [40]. Previous studies reported that the EAI lineage was associated with notably low clustering rates, suggesting they are less likely to be transmitted [40, 41].

The transmission-rate in the remaining provinces was lower (10–21%) compared to WC and EC. The clusters were mostly small with few medium clusters in NW, KZN and GP. The cluster LAM4 with 31 cases were mainly detected in KZN (*n* = 13) and GP (*n* = 9) (Table 4). This cluster is likely the same as the F15/LAM4/KZN strain, previously described as endemic in KZN [2, 24].

The exact drivers of higher rates of transmission in some provinces (districts) over others is less understood. In WC and EC, the high prevalence of Beijing genotype may play a role in driving transmission. The Beijing family have most often been associated with transmission of DR-TB in WC and EC [15, 17, 36]. Lack of ventilation due to the cold weather condition in WC was also reported to contribute to transmission. Other possible drivers of transmission include: population density, socio-economic factors (overcrowded living conditions, patterns of congregation and social mixing, public transportation), high HIV prevalence, and inadequate TB control program (inappropriate or non-compliance to treatment, lack of surveillance, diagnostic and treatment delay). Thus, appropriately targeted interventions based on a better understanding of the drivers of RR-TB transmission at district level is needed for designing successful control measures. This could be accomplished by strengthening district-level health systems and collect additional data from patients in order to identify risk factors that facilitate transmission.

This study has several limitations. Firstly, there is a selection bias in the study population because only culture positive samples in selected districts/provinces were included. Also, the surveillance system included patients who accessed health care, thus patients undiagnosed and/or died in the community would not be included. As a result, our findings may not be generalizable to the entire South African population. Secondly, sample collection in the different provinces occurred during different time periods, which could have impacted clustering analysis. Areas that had shorter sampling durations may have missed transmission events and underestimated clustering. Thirdly, the epidemiological data needed to support patient-to-patient transmission within genotypic clusters were not available for this analysis. As cases that share a molecular cluster may also reflect common endemic strains. Lastly, the possibility of overestimating clustering and recent TB transmission-rates is possible considering that the basis of the clustering analysis was done using traditional typing, whereas WGS could have offered a better resolution of strains and further discrimination between individuals in clusters. Despite these limitations, our study provides important information on the circulating *RR-TB* strains and potential transmission hotspots in SA.

## Conclusions

Our study provides the first broad insight into RR-TB population structure and transmission in SA. Distinct distribution in RR-TB genotypes was observed in this study, highlighting the need for geographically targeted intervention as well as further research to understand the reasons for such local expansions with specific genotypes. The higher prevalence of EAI1_SOM genotype in MP is of concern requiring further investigation. Large clusters contribute to the burden of RR-TB in WC, EC and MP, highlighting the need for community-wide interventions that decrease transmission.

The high proportion of small clusters identified in the study suggest close contact transmission events, emphasizing the importance of contact case investigations and infection control as the primary intervention in SA. It highlights the urgent need for implementation of World Health Organization (WHO) and National Department of Health (NDOH) guidelines regarding the treatment of infection with TB preventative therapy for all high-risk contacts exposed to RR-TB at the household level. This will help in reducing household transmission thus reducing the burden of morbidity and mortality as a result of TB.

## Methods

### Study population and setting

The surveillance included patients newly diagnosed with RR-TB via Xpert MTB/RIF assay between 2014 and 2018. The surveillance was implemented at eight of the nine provinces, with at least one district targeted per province. In 2014, the surveillance was initiated in the following districts (provinces): Nelson Mandela Metro (Eastern Cape [EC]), Frances Baard (Northern Cape [NC]), Ehlanzeni (Mpumalanga [MP]), Dr. Kenneth Kaunda (North West [NW]) and Umgungunglovo (*KwaZulu*-*Natal [KZN]*). The surveillance was further expanded to the City of Johannesburg (Gauteng [GP]) in 2015, Mangauang (Free State [FS]) in 2016 and City of Cape Town Metro, Cape Winelands and West Coast (Western Cape [WC]) in 2017. Based on operational and feasibility issues, some of the districts were limited to sentinel hospitals with feeder clinics while for some districts the surveillance covered all facilities. The staggered timelines was in part due to the implementation considerations (approvals, logistics etc.) and new areas were added sequentially. Additionally, the health system operates differently in each area and these also impacted on how the surveillance was set up.

All RR-TB samples were submitted to CTB, NICD for culture and genotyping. All the methods were carried out in accordance with relevant guidelines.

### Genotyping methods and analysis

All culture confirmed samples were genotyped by combination of spoligotyping and 24-loci MIRU-VNTR typing. Spoligotyping was performed using the international standardized method [10] and patterns were analysed and classified by shared-international-type (SIT) in accordance with the Fourth-International-Spoligotyping-Database [20]. Standard 24-loci MIRU-VNTR typing was performed using the commercial kit (Genoscreen, Lille, France) and 24-capillary ABI 3500xl genetic analyzer (Applied Biosystems, California, USA) as described by the manufacturer. Sizing of PCR fragments and MIRU-VNTR allele assignation were performed using GeneMapper software 5.0 (Applied Biosystems).

### Cluster definition and analysis

A genotype cluster was defined as two or more patients having identical patterns by both spoligotyping and 24-MIRU-VNTR typing. A non-clustered (unique) case was defined as any case from the study population having a unique pattern not shared by any other case. The proportion of cases attributed to recent TB transmission (transmission-rate) was calculated by the n-1 method according to the formula: (n_c_ − c)/n in which n = total number of cases in the sample, c = is the number of clusters (genotypes represented by at least two cases) and n_c_ = is the total number of cases in a cluster of two or more [42]. The genotype diversity was also calculated (diversity = number of SITs divided by the total number of isolates).

Descriptive statistics were used to present the number and proportion of clustered strains and clusters and distribution of cluster size. We defined the size of a cluster by categorising cases into four groups, according to the size of the genotypic cluster: 2–5 cases per cluster [small cluster], 6–10 cases per cluster [medium cluster], 11–25 cases per cluster [large cluster], and ≥ 26 cases [very large cluster].

## Supplementary Information

**Additional file 1.**

## Data Availability

The datasets used and/or analysed during the current study is available as supplementary data.

## Abbreviations

DR-TB: Drug-resistant tuberculosis
SA: South Africa
RR: Rifampicin-Resistant
MIRU-VNTR: Mycobacterial interspersed repetitive-units-variable-number tandem repeats
LAM: Latin American and Mediterranean
EAI1_SOM: East-African-Indian-Somalian
H: Haarlem
*M. tuberculosis*: *Mycobacterium tuberculosis*
RFLP: Restriction fragment length polymorphism
PCR: Polymerase chain reaction
WGS: Whole genome sequencing
CTB: Center for TB
NICD: National Institute for Communicable Diseases
WC: Western Cape
EC: Eastern Cape
NW: North West
KZN: Kwa-Zulu Natal
GP: Gauteng
NC: Northern Cape
FS: Free State
MP: Mpumalanga
SIT: Shared International Type
MDR: Multidrug-resistant
XDR: Extensively drug-resistant
n: Number
